# Occurrence and overlap of physical and mental health conditions in autistic adults

**DOI:** 10.1177/13623613251362346

**Published:** 2025-09-12

**Authors:** Carolien Torenvliet, Tulsi A Radhoe, Hilde M Geurts

**Affiliations:** 1 University of Amsterdam, The Netherlands; 2 Radboud University, The Netherlands; 3 Leo Kannerhuis, Autism Clinic (Youz/Parnassia Group), The Netherlands

**Keywords:** autism, mental health, physical health, psychometric network analysis

## Abstract

Mental and physical health conditions are a major topic of concern in autistic adults, but studies rarely consider their overlap. Therefore, this study assessed physical health conditions and mental health conditions in autistic adults in the Netherlands and potential associations between them. Using questionnaires, we compared autistic (*n* = 327) and non-autistic (*n* = 274) adults (30–90 years) on mental health conditions, physical health conditions, and health-related quality of life. Autistic adults reported lower health-related quality of life than non-autistic adults. Logistic regressions indicated significantly higher rates of all mental health conditions reported by autistic adults, most common being mood (45%), anxiety (22%), and personality disorders (21%), with odds ratios ranging from six to 34. Regarding physical health conditions, autistic adults reported significantly higher rates of bowel conditions (27%), allergies (48%), and hypothyroid conditions (6%), with odds ratios ranging from two to four. Psychometric network analysis of autism and the most frequently occurring mental health conditions and physical health conditions highlighted autism as a central node, followed by mood and personality disorders, bowel and respiratory conditions, and allergies. Mental health conditions were tightly clustered, indicating high comorbidity. While no single condition connected physical and mental health in particular, we found several links between the two. These findings emphasize the need for improved healthcare and broader societal changes to enhance the well-being of autistic individuals.

**Lay Abstract:**

Autistic adults often face a range of physical and mental health conditions, but the relationship between these two types of health issues is not well understood. Our study looked at how often physical and mental health conditions in autistic adults occurred. We also studied the connections between these conditions, using a method called psychometric network analysis. We surveyed 327 autistic and 274 non-autistic adults, aged 30–90 years, about potential health conditions they faced and the perception of the quality of their health, also known as health-related quality of life. We found that autistic adults had a lower health-related quality of life and reported higher rates of all mental health conditions. Mood (45%), anxiety (22%), and personality disorders (21%) were most common. Autistic adults were between six and 34 times more likely to have these mental health conditions compared to non-autistic adults. In terms of physical health, autistic adults reported higher rates of bowel conditions (27%), allergies (48%), hypothyroid conditions (6%), and less robustly of strokes (CVA/TIAs; 3%), and rheumatic conditions (31%)— and a two- to four-times higher risk than non-autistic adults. Using psychometric network analysis, we found that mental health conditions in autistic adults are closely linked, showing how complex their health challenges are. While there was no single condition that connected physical and mental health in particular, we found several links between the two. These findings emphasize the need for improved healthcare and broader societal changes to enhance the well-being of autistic individuals.

## Introduction

Over the past decade, the increased occurrence of physical health conditions (PHCs) in autistic adults has gained attention (Rydzewska et al., 2021). Multiple studies suggest that autistic adults are at risk for PHCs, such as cardiovascular and respiratory diseases (Charlton et al., 2023; Weir et al., 2021), neurological conditions including neurodegenerative disorders and epilepsy (Croen et al., 2015; Hand et al., 2020; Vivanti et al., 2021), bowel conditions, and immune conditions (Hand et al., 2020). Parallel to the increased occurrence of PHCs, mental health conditions (MHCs) are highly prevalent in autistic adults, with mood and anxiety disorders being the most common, alongside elevated rates of schizophrenia and attention deficit/hyperactivity disorder (ADHD; Lai et al., 2019). Given that mortality risks are approximately twice as high in autistic adults (Hirvikoski et al., 2016; Krantz et al., 2023), it is clear that PHCs and MHCs in autistic adults must be taken seriously (Lai, 2023).

As autistic adults seem at risk for developing both PHCs and MHCs, it seems important to gain further insight into the overlap between physical and mental health in autistic adults. A recent overview of the mental health challenges of autistic adults highlighted the relevance of cascading health effects (Lai, 2023). For example, an increased allostatic load caused by lifelong symptoms of anxiety might lead to cardiovascular risk factors such as high blood pressure or diabetes, which in turn might cause cardiovascular or neurodegenerative disease. Such proposed pathways between physical and mental health are increasingly recognized in the general population (Bekhuis et al., 2016; El Baou et al., 2023; Prince et al., 2007; Rødevand et al., 2021). In 2017, the World Health Organization reported on the risks of comorbidity between physical and mental health and the need for implementing collaborative care, in which health professionals from different fields work together to address both aspects of well-being (World Health Organization, 2017). Although the potential benefits of collaborative care for autistic individuals were pointed out recently (Kalb et al., 2023), limited research has been conducted on the overlap between physical and mental health in autistic adults.

Recent studies have begun to address this gap. For example, one study found an association between depression symptoms, emotion regulation, and cardiovascular risk factors (Charlton et al., 2023). Another study showed that anxiety mediated the association between physical symptoms and being autistic (Grant et al., 2022). In addition, two recent studies indicated that psychiatric comorbidity and stress were associated with gastrointestinal symptoms and metabolic conditions in both autistic and non-autistic adults (Warreman, Nooteboom, Leenen, et al., 2023; Warreman, Nooteboom, Terry, et al., 2023).

However, all of these preliminary studies only included a limited selection of PHCs and MHCs, making it hard to conclude on which conditions may play a particularly large role. Therefore, it seems important to study such associations cohesively instead of in isolation. This is especially relevant in light of a recent study that demonstrated increased interrelatedness of PHCs in autistic individuals compared to non-autistic individuals, as shown through psychometric network analysis (Ward et al., 2023). Psychometric network analysis is a method designed to explore the associations of various symptoms, classifications, or traits in one model (Borsboom & Cramer, 2013). Therefore, they are a useful tool to study mental and physical health in autistic adults in a cohesive manner. Moreover, psychometric network enables the identification of “bridge conditions”—conditions that connect physical and mental health challenges, influencing or exacerbating both domains through shared biological or psychosocial pathways (adapted from Cramer et al. (2010) and Jones et al. (2021)). By investigating bridge conditions between mental and physical health clusters, we may be able to clarify which conditions play a particularly large role in the health challenges of autistic adults. Finally, psychometric network analysis allows for the inclusion of covariates, such as sex and age, which may affect MHCs and PHCs as experienced by autistic people (Lai, 2023). Most samples to date consisted of a large proportion of young adults (<30/40 years; Croen et al., 2015; Fortuna et al., 2016; Vivanti et al., 2021; Weir et al., 2021), while physical health problems are likely to be more prominent in older adults (Hand et al., 2020)—usually defined as adults older than 50 (Roestorf et al., 2019). Therefore, addressing the effects of age seems particularly relevant.

Taking into account the findings and gaps to date, this study aims to (1) assess the frequency of self-reported PHCs and MHCs in a large cohort (*n* = 791) of autistic and non-autistic adults and to compare their health-related quality of life (H-QoL), and (2) assess associations between physical and mental health clusters using psychometric network analysis, with autism and bridge conditions as nodes of interest and sex and age as covariates.

## Methods

### Participants

**Figure 1.**
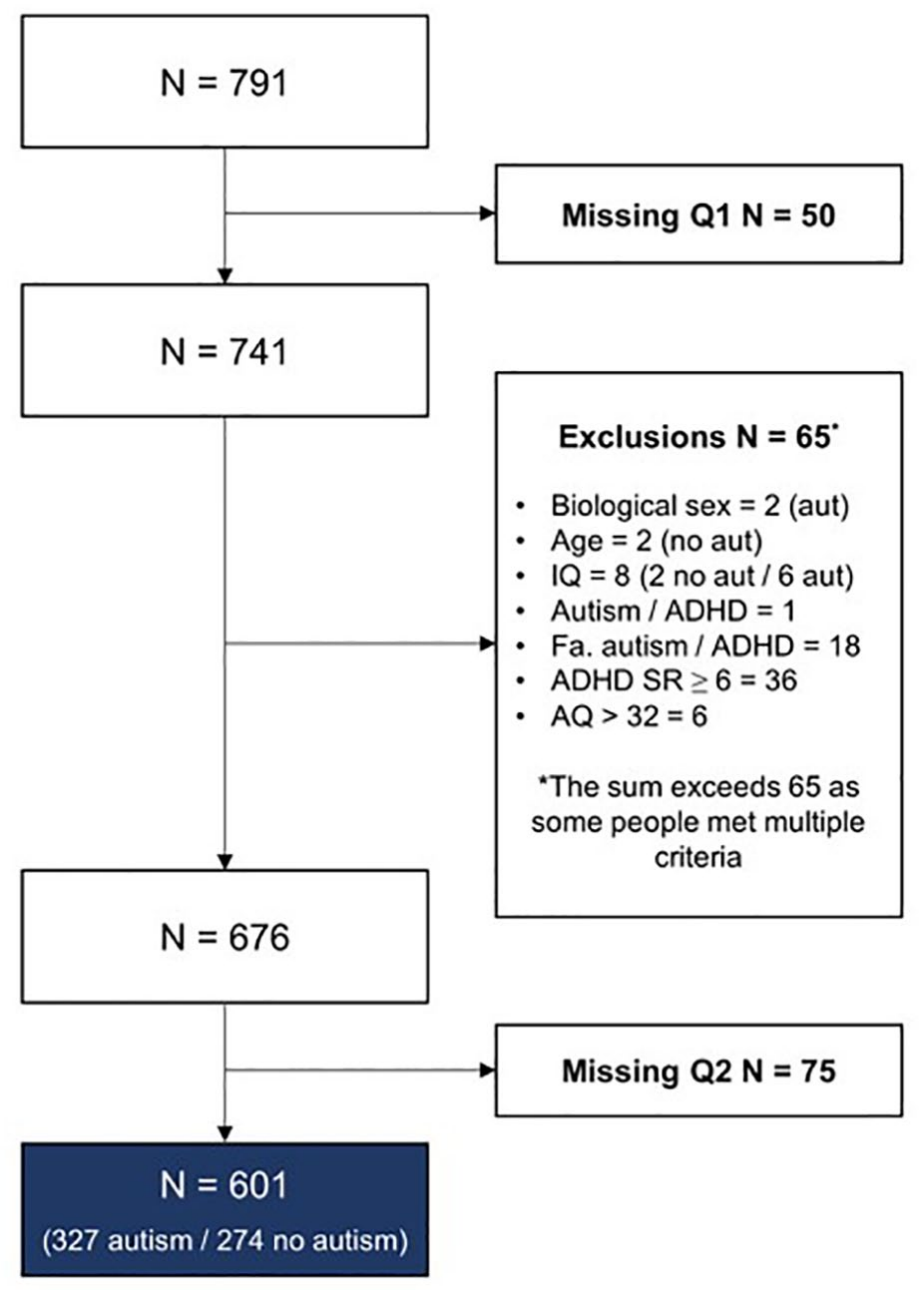
Attrition and exclusion of participants in this study. Aut, autism; no aut, no autism; Fa., familial; Q1, questionnaire booklet 1; Q2, questionnaire booklet 2.

Participants (*n* = 791) in this study were part of a multistage, overlapping longitudinal cohort study on Aging & Autism (Geurts et al., 2021). They were recruited via several clinical institutions across the Netherlands, (social) media advertisements from autism networks, and the social networks of the researchers, research assistants, and students. The advertisements primarily highlighted aging in autistic adults, with no specific emphasis on mental or physical health. In total, 601 participants between 30 and 85 years old were included (*n*_autism_ = 327, *n*_no autism_ = 274); see Figure 1. Exclusion criteria for both groups were (1) age younger than 30 or older than 90 years old, (2) insufficient understanding of Dutch to complete the questionnaires, and/or (3) a self-reported diagnosis of intellectual disability and/or reported IQ < 70. Autistic individuals were also excluded if they reported having no registered clinical diagnosis according to the DSM (*Diagnostic and Statistical Manual of Mental Disorders*; American Psychiatric Association, 2013). Additional exclusion criteria for the non-autistic group were (1) a history of autism, 2() a history of AD(H)D, (3) a history of having more than one psychosis, (4) first-degree relatives (i.e. parents, children, siblings) with autism and/or AD(H)D, (5) Autism Spectrum Quotient (AQ; Baron-Cohen et al., 2001) >32, and/or (6) ADHD Rating Scale (ADHD-SR; Kooij et al., 2005) ⩾6. These exclusion criteria were applied to minimize the chance of including undiagnosed autistic participants in the non-autistic group.

### Materials

*H-QoL* was measured by the World Health Organization Quality of Life Questionnaire-BREF (WHO-QoL-BREF; Whoqol Group, 1998). The WHO-QoL-BREF consists of 26 items, each rated on a 5-point scale to indicate how participants felt during the past 2 weeks. We used their perceived overall health on a scale from 1 (very poor H-QoL) to 5 (excellent H-QoL) and computed scores on the physical health subscale. This subscale was based on seven questions with subscale scores ranging from 4 (very poor H-QoL) to 20 (excellent H-QoL). Scores were mean imputed if less than two questions on this subscale were missing.

*MHCs* were assessed using our in-house developed general questionnaire (Geurts et al., 2021). Specifically, participants were asked about the presence of mood disorders, anxiety disorders, personality disorders, alcohol/drug dependency, or any other (self-described) MHC. Self-described conditions were categorized as any of the aforementioned conditions, eating disorders, post-traumatic stress disorder (PTSD), obsessive-compulsive disorder (OCD), and other MHCs. Categorizations were checked with a second, independent rater. Reports of ‘current’ and ‘past’ diagnoses were combined to reflect lifetime occurrence rates, similar to the operationalization of PHCs.

*PHCs* were assessed using the Health Questionnaire (HQ; Central Bureau of Statistics (CBS), 1989). The HQ included 15 categories of PHCs, which we grouped based on the questionnaire’s structure: respiratory conditions, cardiovascular conditions and diabetes, rheumatic conditions, cancer, ulcer, bowel syndromes, liver diseases, epilepsy, allergies, thyroid disease, or any other (self-described) PHC. Self-described conditions were categorized as any of the aforementioned conditions, high cholesterol, obesity, autoimmune disease, headache/migraine, central nervous system (CNS) and degenerative diseases (e.g. Parkinson’s, multiple sclerosis (MS)), dementia, insomnia, nutrition-related conditions (vitamin deficiency), and other PHCs. This categorization follows our questionnaire as well as the most commonly reported other classifications in previous studies (e.g. Hand et al., 2020). Categorizations were checked with a second, independent rater. Reports of all diagnoses reflected lifetime occurrence rates. In addition, the HQ included two questions prompting participants to rate their overall physical health and an age-relative comparison using a 1 (very poor) to 5 (very good) scale.

### Procedure

The study was part of a larger longitudinal study on Aging & Autism (see for details: Geurts et al. (2021). All data used for this study were part of Wave 3 (2018–2020). These data were also published in Radhoe et al. (2023), but with different operationalizations (only sum scores) and purposes (subgroup analysis). Participants first completed written informed consent, after which they filled in the first series of questionnaires (Q1) that contained all questions regarding the exclusion criteria (general questionnaire, AQ, ADHD-SR) and their history of MHCs (general questionnaire). Participants that could be included were sent a second series of questionnaires (Q2) that included the HQ and WHO-QoL BREF. Questionnaires could be completed online or on paper according to the participant’s preference. Participants received €7.50 for their participation. The study was approved by the ethical review board of the Department of Psychology of the University of Amsterdam (2018-BC-9285) and performed according to the principles of the Declaration of Helsinki.

### Community involvement statement

We worked together with a group of four older and/or autistic adults (also referred to as the think tank) for our overall study on Aging & Autism (Geurts et al., 2021). We met with the think tank at least three times a year (either online or in person) to discuss the recruitment strategies, information for participants, the interpretation of study results and other study-related matters. The members were paid for their contributions. Since the overall study had already been completed, they were not engaged in the design or outcomes of this study.

## Analyses

All analyses were performed in RStudio, version 4.2.1 (RStudio Team, 2020). Our preregistered analysis plan (https://aspredicted.org/R1X_QX2) consisted of four steps: first, we calculated differences in H-QoL and self-rated health between autistic and non-autistic individuals (independent *t*-test). We also calculated rates of MHCs and PHCs in autistic and non-autistic adults, and estimated (un)corrected (age, sex, education level) odds ratios (ORs) and their 95% confidence interval (CI) using separate logistic regressions for each condition. As we may have ended up with low frequencies in some cells of the contingency tables, we employed Firth’s biased-reduced logistic regression using the “*logistf*” package (Heinze & Schemper, 2002). We reported significant differences (*p* < 0.05) after correcting for family-wise errors (Benjamini & Hochberg, 1995). Second, we calculated rates and (corrected) ORs for older participants only (50+). Third, we fitted an Ising model with autism and the five most frequently occurring PHCs and four most frequently occurring MHCs as nodes (10). Fourth, we added age (50+/50−) and sex as covariates in a second step.

For the psychometric network analysis, we followed the suggested guidelines in Burger et al. (2022). We used the *IsingFit* package (Van Borkulo et al., 2014) within the *bootnet* wrapper (Epskamp et al., 2018), applying the extended least absolute shrinkage and selection operator (eLASSO) to impose a regularization penalty and control for spurious associations. Model selection was based on the Extended Bayesian Information Criterion (EBIC), which determined the optimal value of the tuning parameter λ. We chose to err on the side of exploration, starting by applying hyperparameter *γ* = 0 and the OR rule—expecting to ensure relatively high sensitivity (0.65–0.76) while maintaining high specificity (0.85–0.98) in our expected sample size of *n_min_* *=* 500 and 10 nodes (see Van Borkulo et al., 2014). Sensitivity analyses were performed to see whether these prespecified parameters are indeed most suitable given the specifics of the network. The *qgraph* package (Epskamp et al., 2012) was used for visualization, with no specific min/max/cut values. We obtained edge weights and their non-parametric bootstrapped CIs with 1000 samples using the *bootnet* package. General width and the overlap of the CIs provided an estimate of the stability and accuracy of the edge weights. We were especially interested in edges directly associated with the autism node. We obtained strength centrality and expected influence from the *bootnet* package to assess the most strongly connected nodes in the network (Bringmann et al., 2019; Robinaugh et al., 2016), with a particular focus on the autism node. We obtained bridge centrality from the *networktools* package to identify potential bridge conditions between MHCs and PHCs (Jones et al., 2021). Centrality stability was obtained from the CS-coefficient as well as CIs using case-drop bootstrapping (1000 samples). The CS-coefficient should preferably be above 0.5, but minimally above 0.25 (Epskamp et al., 2018). Of note, Ising models cannot handle missing data, therefore only complete cases were used for all analyses (see Figure 1).

## Results

The total sample consisted of 601 participants (*n*_autism_ = 327, *n*_no autism_ = 274). Table 1 provides group comparisons on descriptive variables, self-rated health, and H-QoL. The non-autistic group was significantly older than the autistic group. Therefore, adjusted ORs were regarded as our primary outcome. The non-autistic group consisted of significantly more individuals from non-Dutch backgrounds than the autistic group, yet correcting for this did not change any of our outcomes. Therefore, the results are presented as preregistered. Other descriptive differences (i.e. AQ, ADHD-SR) were expected given our exclusion criteria. Self-rated health and H-QoL were significantly lower in the autistic compared to the non-autistic group. Effect sizes ranged from medium to large.

**Table 1.** Group comparisons on descriptives, self-rated health, and H-QoL.

	Autism (*n* *=* 327)	No autism (*n* = 274)	*χ*^2^-value	
	*N* (%)	*N* (%)		
Sex^a^			0.90	
Male	170 (52)	154 (56)		
Female	157 (48)	120 (44)		
Education^b^			2.08	
Jr. sec./Practical	13 (6)	10 (5)		
Sr. sec./Voc.	122 (67)	172 (63)		
University degree	51 (26)	92 (32)		
Employment^c^			58.96**	
No employment	183 (56)	77 (28)		
Part-time	83 (26)	78 (28)		
Full-time	59 (18)	118 (43)		
Background^d^			7.85**	
Dutch	298 (91)	228 (83)		
Non-Dutch	29 (9)	46 (17)		
	Mean (SD), range	Mean (SD), range	*t*-value	Cohen’s *d*
Age in years	52.86 (12.56), 30–84	56.47 (13.73), 30–85	−3.34**	−0.28
AQ total^e^	34.41 (7.60), 10–48	13.62 (5.96), 2–31	37.57**	3.01
Att ADHD-SR^f^	2.94 (2.48), 0–9	0.46 (0.99), 0–5	16.49**	1.27
HI ADHD-SR^g^	3.24 (2.25), 0–9	0.82 (1.12), 0–5	17.02**	1.33
Health rate self^h^	3.61 (0.79), 1–5	4.14 (0.66), 1–5	−8.76**	−0.71
Health rate other^i^	2.86 (1.02), 1–5	3.48 (0.92), 1–5	−7.82**	−0.63
H-QoL overall^j^	3.12 (1.03), 1–5	3.96 (0.86), 1–5	−10.98**	−0.89
H-QoL subscale^k^	13.31 (2.79), 6–20	16.46 (2.28), 7–20	−15.24**	−1.23

Jr. sec./Practical: Junior secondary or practical education, Sr. sec/voc: Senior secondary education or vocational college SD: standard deviation.

a Biological sex described as other was provided as an option, but did not occur in the current sample.

b Level of education was determined by the Verhage Coding System (Verhage, 1964).

c We did not distinguish between unemployment and retirement.

d Dutch background was based on whether participants’ parents were born outside the Netherlands.

e AQ total reflects the total score on the Autism Spectrum Quotient (AQ; Baron-Cohen et al., 2001).

f Att ADHD-SR reflects the total score on the attention subscale of the Attention-Deficit Hyperactivity Disorder—Rating Scale (ADHD-SR) in adulthood (Kooij et al., 2005).

g HI ADHD-SR reflects the total score on the hyperactivity/impulsivity subscale of the ADHD-SR in adulthood (Kooij et al., 2005).

h Participants gave a rating (1–5) on their perceived health on the Health Questionnaire (Central Bureau of Statistics (CBS), 1989).

i Participants gave an age-relative rating (1–5) on their perceived health compared to others of their age on the HQ (Central Bureau of Statistics (CBS), 1989).

j Participants’ rating on perceived health on the World Health Organization Quality of Life questionnaire (WHO-QoL; Whoqol Group, 1998)

k Participants’ subscore on the subscale physical health of the WHO-QoL questionnaire (Whoqol Group, 1998).

** *p* < 0.01.

The 50+ subsample consisted of 353 participants (*n*_autism_ = 191, *n*_no autism_ = 162) and is described in Table S1. Characteristics and group differences in the 50+ subsample mimic those of the total sample.

### Occurrence rates and ORs of MHCs

Occurrence rates and ORs for all MHCs are provided in Table 2. Overall, the four most frequently reported conditions were mood disorders (*n* *=* 156, 26%), anxiety disorders (*n* *=* 79, 13%), personality disorders (72, 12%), and PTSD (19, 3%). Autistic adults reported all MHCs significantly more frequently than non-autistic adults, with adjusted ORs ranging from 5 (drug addiction) to 30 (PTSD). Table S2 provides occurrence rates and ORs for all MHCs in the 50+ subsample. Although reports of MHCs were generally lower in these older groups, and particularly in the autism group, the pattern of results was fairly similar. Adjusted ORs failed to reach significance for eating disorders and OCD within this subsample.

**Table 2.** Occurrence rates and odds ratios of mental health conditions in autistic adults.

Condition	Autism	No autism	OR	CI	χ^2^	Adj. OR	Adj. CI	Adj. χ^2^
Anxiety disorders	71 (21.7%)	8 (2.9%)	8.74	4.44–19.53	52.25**	8.05	4.07–18.05	46.59**
Mood disorders	146 (44.6%)	10 (3.6%)	20.33	11.07–41.48	Inf**	20.19	10.88–41.56	Inf**
Personality disorders	70 (21.4%)	2 (0.7%)	29.84	10.17–144.73	Inf**	27.52	9.33–133.77	68.76**
Alcohol addiction	9 (2.8%)	0 (0%)	16.38	2.06–2114.58	8.50**	14.23	1.79–1837.45	7.37**
Drug addiction	10 (3.1%)	1 (0.4%)	6.03	1.40–56.12	6.21*	5.17	1.19–48.41	4.95*
Eating disorders^a^	7 (2.1%)	0 (0%)	12.85	1.55–1670.13	6.25*	10.85	1.3–1411.67	5.19*
Post-traumatic stress disorder^a^	19 (5.8%)	0 (0%)	34.70	4.73–4423.16	20.37**	30.01	4.07–3828.52	17.72**
Obsessive compulsive disorder^a^	5 (1.5%)	0 (0%)	9.36	1.05–1231.15	4.07*	9.82	1.09–1296.98	4.21*
Other^b^	20 (6.1%)	1 (0.4%)	12.16	3.09–109.93	16.87**	10.59	2.68–96	14.40**

OR: odds ratio; CI: confidence interval; Adj.: adjusted for age, sex, and educational level; pr: pressure; dis: disease; Inf: infinite.

a These conditions/diseases were not specifically asked but named spontaneously. Therefore, occurrence rates of these categories should be interpreted with care.

b Other conditions that were spontaneously named varied widely and could not be categorized.

* Corrected *p* < 0.05; **Corrected *p* < 0.01.

### Occurrence rates and ORs of PHCs

Occurrence rates and ORs for all PHCs are provided in Table 3. Overall, the five most frequently reported conditions were allergies (*n* = 232, 39%), rheumatic conditions (*n* = 177, 29%), cardiovascular conditions (*n* *=* 146, 24%), bowel conditions (*n* = 124, 20%), and respiratory conditions (*n* = 60, 10%). After adjusting for age, sex, and education, autistic adults reported some PHCs significantly more frequently than non-autistic adults, being total bowel conditions and obstipation, total allergies and eczema, total thyroid conditions and hypothyroid, and other PHCs (see Table 3). Adjusted ORs of these conditions ranged from 2.29 (eczema) to 3.97 (obstipation). Although autistic adults reported somewhat more Cerebrovascular Accidents (CVA) and/or Transient Ischemic Attacks (TIAs), rheumatic conditions, and irritable bowel syndromes (IBS) than non-autistic adults the differences were not statistically significant after controlling for multiple testing (see Table 3). Table S3 provides occurrence rates and ORs for all PHCs in the 50+ subsample. Occurrence of PHCs was stable or somewhat higher in both groups (autism/no autism).

**Table 3.** Occurrence rates and odds ratios of physical health conditions in autistic adults.

Condition		Autism	No autism	OR	CI	χ^2^	Adj. OR	Adj. CI	Adj. χ^2^
Respiratory	Total^a^	37 (11.3%)	23 (8.4%)	1.38	0.81–2.4	1.39	1.27	0.73–2.22	0.71
	Asthma	26 (8%)	19 (6.9%)	1.15	0.63–2.14	0.21	1.01	0.54–1.89	<0.01
	COPD	14 (4.3%)	5 (1.8%)	2.27	0.88–6.72	2.86	2.47	0.94–7.47	3.33
Cardiovascular	Total	71 (21.7%)	75 (27.4%)	0.74	0.51–1.07	2.59	0.95	0.63–1.42	0.07
	Heart attack	27 (8.3%)	33 (12%)	0.66	0.39–1.12	2.36	0.81	0.46–1.43	0.52
	CVA/TIA	11 (3.4%)	3 (1.1%)	2.82	0.92–11.16	3.28	3.76	1.19–15.3	5.17^†^
	Blood pr.	43 (13.1%)	48 (17.5%)	0.71	0.46–1.11	2.20	0.92	0.57–1.48	0.13
	Vascular dis.	12 (3.7%)	7 (2.6%)	1.41	0.57–3.72	0.56	1.71	0.68–4.61	1.29
	Diabetes	26 (8%)	14 (5.1%)	1.58	0.83–3.13	1.89	1.85	0.95–3.74	3.31
Rheumatic	Total	102 (31.2%)	75 (27.4%)	1.20	0.84–1.71	1.04	1.49	1.02–2.19	4.28^†^
	Fibromyalgia	15 (4.6%)	8 (2.9%)	1.56	0.68–3.82	1.07	1.44	0.6–3.66	0.65
	SLE	0 (0%)	1 (0.4%)	0.28	0.00–5.24	0.72	0.23	0.00–4.53	0.93
	Arthritis	83 (25.4%)	69 (25.2%)	1.01	0.70–1.46	<0.01	1.32	0.89–1.99	1.90
Cancer		25 (7.6%)	22 (8%)	0.95	0.52–1.72	0.03	1.13	0.61–2.1	0.15
Ulcer		14 (4.3%)	7 (2.6%)	1.65	0.69–4.27	1.25	1.98	0.8–5.35	2.15
Bowel	Total	89 (27.2%)	35 (12.8%)	2.53	1.66–3.92**	19.45	2.46	1.6–3.84	17.34**
	IBS	43 (13.1%)	17 (6.2%)	2.25	1.28–4.11*	8.13	2.00	1.12–3.71	5.54^†^
	Obstipation	49 (15%)	11 (4%)	4.07	2.17–8.28**	21.26	3.97	2.09–8.15	19.62**
	Crohn’s dis.	1 (0.3%)	2 (0.7%)	0.50	0.05–3.79	0.46	0.57	0.05–4.3	0.30
Liver	Total	5 (1.5%)	2 (0.7%)	1.86	0.44–10.41	0.70	1.79	0.42–10.03	0.60
	Cirrhosis	0 (0%)	1 (0.4%)	0.28	0.00–5.24	0.72	0.36	0.00–6.6	0.45
	Hepatitis	2 (0.6%)	1 (0.4%)	1.40	0.19–15.37	0.11	1.34	0.18–14.74	0.08
Epilepsy		6 (1.8%)	1 (0.4%)	3.69	0.77–35.52	2.58	3.86	0.80–37.41	2.75
Allergy	Total	158 (48.3%)	74 (27%)	2.52	1.79–3.56	28.91**	2.41	1.71–3.42	25.61**
	Hay fever	82 (25.1%)	51 (18.6%)	1.46	0.99–2.17	3.6	1.35	0.91–2.01	2.16
	Eczema	72 (22%)	29 (10.6%)	2.36	1.5–3.8	14.24**	2.29	1.45–3.7	12.95**
Thyroid	Total	24 (7.3%)	9 (3.3%)	2.26	1.08–5.1	4.73^†^	2.96	1.32–7.19	7.14**
	Graves’ dis.	0 (0%)	1 (0.4%)	0.28	0.00–5.24	0.72	0.82	0–83.35	0.01
	Hypothyroid	19 (5.8%)	5 (1.8%)	3.10	1.26–8.96	6.28^†^	3.78	1.44–11.69	7.54**
	Hyperthyroid	1 (0.3%)	1 (0.4%)	0.84	0.07–10.36	0.02	0.89	0.07–11.49	0.01
Auto-immune conditions^b^		7 (2.1%)	1 (0.4%)	4.27	0.93–40.63	3.42	4.1	0.88–39.22	3.15
Headache/Migraine^b^		9 (2.8%)	5 (1.8%)	1.46	0.52–4.55	0.50	1.13	0.39–3.56	0.05
Neurological and CNS disease^b^		5 (1.5%)	4 (1.5%)	1.03	0.29–3.85	<0.01	0.94	0.26–3.6	0.01
Sleep disorders^b^		6 (1.8%)	1 (0.4%)	3.69	0.77–35.52	2.58	3.77	0.78–36.55	2.65
Nutrition deficiencies^b^		1 (0.3%)	0 (0%)	2.52	0.13–368.65	0.36	3.48	0.15–606.88	0.57
Other^c^		50 (15.3%)	18 (6.6%)	2.52	1.47–4.52	11.59**	2.68	1.54–4.86	12.53**

OR, odds ratio; CI, confidence interval; Adj, adjusted for age, sex and educational level; pr, pressure; dis, disease; Inf, infinite.

a Total classifications included not only the specific conditions that are specified as sub-conditions, but also other unspecified/unknown conditions within that category.

b These conditions/diseases were not specifically asked but named spontaneously. Therefore, occurrence rates of these categories should be interpreted with care.

c Other conditions that were spontaneously named varied widely and could not be categorized.

* Corrected *p* < 0.05; **Corrected *p* < 0.01. ^†^Differences did not persist after correcting for family wise errors (Benjamini & Hochberg, 1995).

### Network structure of autism, PHCs, and MHCs

**Figure 2.**
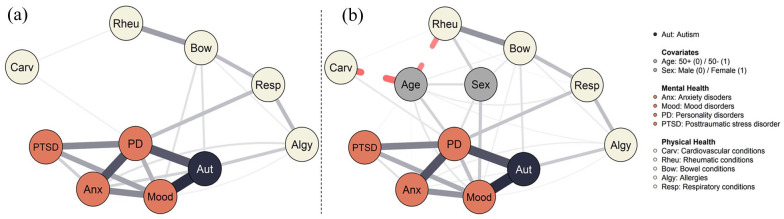
Psychometric network of autism, mental, and physical health (a) and including age and sex as covariates (b). Psychometric networks of autism, mental, and physical health. The legend on the right is applicable to both graphs. Edges represent regularized log-linear relations. Dotted red lines represent negative edges; solid gray lines represent positive edges. Positive associations indicate the presence of a condition, younger age, and female sex.

The resulting network structure of autism, four most prevalent MHCs, and five most prevalent PHCs is displayed in Figure 2(a). The layout was generated using the Fruchterman–Reingold algorithm (Fruchterman & Reingold, 1991), without any specific cut, minimum, or maximum values. Forty-two of the 90 edges were statistically significant and had an average edge weight of 0.34. The minimum edge weight was 0.15 (mood disorders-allergies) and the maximum edge weight was 2.09 (autism-mood disorders). Non-parametric bootstrapping shows fairly wide estimates of the edges (see Figure S1), so although all present edges were significantly different from zero, differences in edge strengths must be interpreted with care. Centrality indices are provided in Figure S2. Case-drop bootstrapping is shown in Figure S3 and indicate stable indices (CS-coefficients between 0.60 and 0.75).

As visualized in Figure 2(a), the network consisted of a tight cluster (>2 connections) of MHCs, together with autism, and a more loosely connected cluster (⩽2 connections) of PHCs. This was substantiated by larger centrality indices for MHCs than PHCs (see Figure S2). Autism was among the most connected nodes in the network, next to personality disorders and mood disorders. These three nodes also formed the clearest bridges with PHCs. Among PHCs, bowel conditions, allergies, and respiratory conditions displayed more (bridge) connections than cardiovascular and rheumatic conditions. Overall, the network featured several separate connections between the MHC and PHC clusters rather than a single condition. Sensitivity analyses (Figure S4) indicated that edges between bowel conditions and mood disorders, bowel conditions and autism, respiratory conditions and personality disorders, and allergies and autism formed the most stable bridges between the clusters.

The resulting network including two covariates (sex and age) is displayed in Figure 2(b). To ensure comparability, the layout of the covariate network was fixed on its previous configuration, with the extra nodes inserted at specified locations. Seventy-two out of the 132 edges were statistically significant and had an average weight of 0.28. Incorporating covariates did not impact the existing associations within the network, except for introducing a significant edge between rheumatic and respiratory conditions. Many nodes (MHCs and PHCs) were linked to age and sex, suggesting the relevance of these covariates to mental and physical health—albeit not altering their interrelationships. Being younger was associated with all MHCs, respiratory conditions, allergies, and bowel conditions, whereas being older was associated with cardiovascular and rheumatic conditions. Female sex was associated with mood and personality disorders, and bowel and rheumatic conditions, whereas male sex displayed no such associations. Sensitivity analyses (Figure S5) showed similar indications. Non-parametric bootstrapped edge weights, central stability indices, and their stability are provided in Supplementary Figures S6–S8. Centrality indices were, again, satisfactory (CS-coefficients > .50).

## Discussion

This study provides additional evidence for an increased vulnerability to PHCs and MHCs in autistic adults. Autistic adults reported lower H-QoL compared to non-autistic adults. Mood disorders occurred in 44% of our autistic sample, and 21% reported diagnoses of anxiety or personality disorders. Autistic adults had a 6- to 34-fold higher risk of reporting mental health diagnoses compared to non-autistic adults. In the physical health domain, the odds were not as high as mental health diagnoses, yet autistic adults reported hypothyroid conditions (6%), bowel conditions (27%), and allergies (48%) more frequently, with odds ratios ranging from approximately two to four. Psychometric network analysis of autism and the most frequently occurring MHCs and PHCs highlighted autism as a central node. Therefore, our study substantiates previously observed health risks for autistic adults.

We observed increased rates of all MHCs and particularly mood disorders, anxiety disorders, and personality disorders. Mood disorders occurred more often than previous meta-analytic evidence suggests (44% vs 11% in Lai et al. (2019)). However, this aligns with higher rates reported in adults and in individuals of the female sex, which reflects the composition of our sample. Estimates of personality disorders were also relatively high, but these estimates vary widely across samples and applied instruments (Rinaldi et al., 2021), and the observed lifetime occurrence rate falls within that range. Within the physical health domain, conditions that showed increased incidence are in line with previous findings suggesting increased risks for gastrointestinal conditions and allergies (Croen et al., 2015; Fortuna et al., 2016; Grant et al., 2022; Hand et al., 2020). The increased incidence of hypothyroid was in line with Hand et al. (2020), yet in contrast to Fortuna et al. (2016) who observed nearly equivalent rates of hypothyroid between autistic and non-autistic adults. For other PHCs, such as cardiovascular conditions, rheumatic conditions, neurodegenerative disorders, epilepsy, migraine/headaches, and immune conditions, we observed lower risks than commonly reported (Croen et al., 2015; Fortuna et al., 2016; Grant et al., 2022; Hand et al., 2020). Although the current findings suggest that the risks for these conditions may be lower than anticipated, the discrepancies across studies emphasize the importance of accounting for methodological and population differences between studies—particularly for conditions with low base rates. More specifically, one explanation for the observed discrepancies is that our results are based on a Dutch convenience and voluntary response sample, whereas most prior research has relied on US epidemiological (insurance) databases. Nonetheless, all findings underscore the health risks faced by autistic adults and the need for appropriate care.

Psychometric network analysis provided further insights on which specific health conditions may warrant targeted support. Autism was central and formed a cluster with the mental health domain. This corroborates with the aforementioned increased occurrence rates and odds for MHCs. Although associations between autism and the physical health domain were also present, our findings emphasize that these associations seem stronger and more complex in the mental health domain. In particular, the connections between all MHCs are in line with previous observations of the multifaceted nature of mental health problems in autistic adults (Lai, 2023) and highlight their complexity. These connections were unchanged after adding sex and age as covariates to the network, although female sex and younger age seemed generally associated with more MHCs and PHCs.

Another characteristic of our networks is that they featured several separate connections between physical and mental health rather than a single bridge condition. In line with Charlton et al. (2023), who found an association between difficulties with emotion regulation and cardiovascular risk factors, broader aspects of emotion regulation may play a greater role in the mental and physical health of autistic adults than any single condition. Given the multifaceted nature of MHCs in autistic adults and the variety of pathways between physical and mental health, it may be promising to incorporate elements of holistic healthcare, such as exercise, yoga, and meditation, into existing interventions to promote physical health while also focusing on managing emotions (Sefen et al., 2020; Stecher et al., 2023). At the same time, it is crucial to acknowledge that the health outcomes of autistic adults are also shaped by societal barriers and systemic inequalities. Factors such as (minority) stress, stigma, camouflaging, loneliness, and socioeconomic disadvantage seem to influence health outcomes (Botha & Frost, 2020; Cage et al., 2018; Moseley et al., 2021; Shaw et al., 2024; Zhuang et al., 2023). Thus, while holistic healthcare approaches may be beneficial at the individual level, they must be accompanied by structural changes in our society to effectively support the well-being of autistic individuals.

Besides these global features of the networks, they drew attention to a few central nodes. In the mental health domain, mood disorders connected to autism, MHCs, and PHCs. In non-autistic adults, it has been shown that psychotherapy aimed at targeting mood disorders might reduce physical health risks, particularly cardiovascular disorders (El Baou et al., 2023). Even though our study showed no such direct indication, mood disorders were associated to both bowel conditions and allergies, which may cascade to poor health in other systems (Ward et al., 2023). Given that mood disorders were reported by 44% of the autistic participants, targeting mood disorders seems to remain a priority in improving autistic adults’ health. In addition, personality disorders were associated with autism and mental health, and formed a bridge to the physical health domain. It is hard to specify what these personality disorders entail, as no subclassifications were recorded in our study. Nonetheless, the centrality of personality disorders is consistent with the idea that those autistic adults with marked personality traits have complex needs that are poorly met (Gillett et al., 2023; Klang et al., 2022; Rydén et al., 2008).

In the physical health domain, bowel conditions, respiratory conditions, and allergies showed most connections to other conditions in the network, suggesting they may be key targets for intervention. Hypothetically, they add to the stress that autistic adults already face and deteriorate other physical and mental health problems (Grant et al., 2022). Evidently, such conditions might also be a consequence of stress and MHCs (Ohrnberger et al., 2017). In both cases, improved medical care might reduce the burden for autistic adults. As noted by others, it is essential to take away existing healthcare barriers (Malik-Soni et al., 2022; Mason et al., 2019; Walsh et al., 2020; Warreman, Ester, et al., 2023). The SPACE framework (Sensory needs, Predictability, Acceptance, Communication, and Empathy) highlights principles for making healthcare more accommodating (Doherty et al., 2023). First steps can be in simple solutions such as increasing consultation time to adapt to longer processing time, securing consistent healthcare providers to accommodate a need for consistency and familiarity, and embedding e-Health solutions to ease communication (Mason et al., 2019; Warreman, Ester, et al., 2023). In addition, addressing the “triple empathy problem”—the mutual misunderstandings between autistic individuals and healthcare providers—might reduce healthcare avoidance and improve interactions (Shaw et al., 2024).

### Strengths and limitations

This study provides insight into the physical and mental health of autistic adults in several ways. First, the large and systematically collected dataset provides a valuable basis for our conclusions. Second, we provided scientific rigor by preregistering the analyses. Third, the use of psychometric network analysis provided novel insights into the centrality of autism in health conditions and the overlap between physical and mental health.

Some aspects of this study limit the aforementioned interpretations. First, the representativeness of our sample was limited as our participants were predominantly White, underrepresented those with practical education and excluded individuals with intellectual disabilities. Second, as self-reported lifetime occurrence was used, we risk interpreting misdiagnoses. Given the qualitative (e.g. Au-Yeung et al., 2019) and quantitative (e.g. Fusar-Poli et al., 2022) evidence on mental health misdiagnoses in autistic adults, it could be the case that our life-time occurrence rates are an overestimation of the mental health problems of autistic people. Especially in the case of personality disorders, some might argue that we run into the risk of interpreting misdiagnoses (Gillett et al., 2023). However, even if these past diagnoses are partly incorrect, our results seem to illustrate that such diagnostic information is valuable in laying out the complexity of mental and physical health of autistic adults. Third, since we rely on self-report, it could be that diagnoses were under- or overreported. Particularly, occurrence rates of those conditions that were named spontaneously were interpreted with care, yet no significant group differences were found for these conditions. Fourth, psychometric network analysis is limited to the nodes included in the network (Borsboom et al., 2021). It is likely that other factors, such as the aforementioned systemic injustices and societal barriers, explain or parse out the centrality of autism in the network. Expanding the current covariates (age and sex) to include such factors could provide further insights into the mechanisms of health problems in autistic adults. Detailed information on employment status could be such a factor as financial security is relevant in explaining health disparities (Macintyre et al., 2018). Finally, as our study was cross-sectional in nature, we cannot conclude on any causal relations between physical and mental health. Longitudinal (intervention) studies seem a vital next step to provide further insight in the root causes of these associations. However, our findings also stress the likelihood of multicausality of mental and physical health problems in autistic adults, which should be considered in longitudinal studies.

## Conclusion

In summary, our findings provide further support for increased physical and mental health challenges in autistic adults. Through psychometric network analysis, we visualized the central role of autism in these health challenges, although the role of third factors—such as systemic inequalities and societal barriers—requires further investigation. Rather than identifying a single condition, our results highlight multiple connections between PHCs and MHCs, emphasizing the need for improved healthcare and broader societal changes to enhance the well-being of autistic individuals.

## Supplemental Material

sj-docx-1-aut-10.1177_13623613251362346 – Supplemental material for Occurrence and overlap of physical and mental health conditions in autistic adults Supplemental material, sj-docx-1-aut-10.1177_13623613251362346 for Occurrence and overlap of physical and mental health conditions in autistic adults by Carolien Torenvliet, Tulsi A Radhoe and Hilde M Geurts in Autism
