# Supplementary material for: Occurrence and overlap of physical and mental health conditions in autistic adults
Source: Autism. 2025 Sep 12;29(12):3124–35. doi: 10.1177/13623613251362346 (PMC12618707; doi:10.1177/13623613251362346)
Supplement: sj-docx-1-aut-10.1177_13623613251362346 – Supplemental material for Occurrence and overlap of physical and mental health conditions in autistic adults [file sj-docx-1-aut-10.1177_13623613251362346.docx]

**Table S1.** Group comparisons on descriptives, self-rated health, and H-QoL in the 50+ Subsample.

|  | Autism (*n*=191) | No autism (*n*=192) |  |  |
| --- | --- | --- | --- | --- |
|  | *N* (%) | *N* (%) | χ^2^-value | |
| Sex^a^ |  |  | 0.99 | |
| Male | 118 (62) | 108 (56) |  | |
| Female | 73 (48) | 84 (44) |  | |
| Education^b^ |  |  | 1.27 | |
| Junior secondary or practical education | 12 (6) | 10 (5) |  | |
| Senior secondary education or vocational college | 128 (67) | 121 (63) |  | |
| University degree | 51 (27) | 61 (32) |  | |
| Background^c^ |  |  | 5.27* | |
| Dutch | 178 (93) | 164 (85) |  | |
| Non-Dutch | 13 (7) | 28 (15) |  | |
|  | Mean (SD), range | Mean (SD), range | *t*-value | Cohen’s *d* |
| Age in years | 61.53 (7.92), 50-85 | 63.74 (8.68), 50-85 | -2.60* | -0.27 |
| AQ total^d^ | 34.55 (7.10), 13-48 | 14.16 (5.91), 3-31 | 30.55** | 3.12 |
| Att ADHD-SR^e^ | 2.9 (2.44), 0-9 | 0.43 (0.99), 0-5 | 12.91** | 1.34 |
| HI ADHD-SR^f^ | 3.06 (2.16), 0-9 | 0.67 (1.02), 0-5 | 13.76** | 1.42 |
| Health rate self^g^ | 3.70 (0.80), 1-5 | 4.07 (0.69), 1-5 | -4.80** | -0.49 |
| Health rate other^h^ | 3.06 (1.01), 1-5 | 3.48 (0.94), 1-5 | -4.18** | -0.43 |
| H-QoL overall^i^ | 3.26 (1.04), 1-5 | 3.90 (0.90), 1 -5 | -6.43** | -0.66 |
| H-QoL subscale^j^ | 13.74 (2.85), 7-20 | 16.33 (2.40), 7-20 | -9.60** | -0.98 |

*Note*. M, male; F, female; Y, Yes; N, No; SD, standard deviation; **=*p*<.01; *=*p*<.05.

^a^ Biological sex described as other was provided as an option, but did not occur in the current sample.

^b^ Level of education was determined by the Verhage Coding System (Verhage, 1964).

^c^ Dutch background (yes / no) was based on whether participants parents were born outside the Netherlands

^d^ AQ total reflects the total score on the Autism-spectrum Quotient (AQ; Baron-Cohen et al., 2001).

^e^ Att ADHD-SR reflects the total score on the attention subscale of the Attention-Deficit Hyperactivity Disorder – Rating Scale (ADHD-SR) in adulthood (Kooij et al., 2005).

^f^ HI ADHD-SR reflects the total score on the hyperactivity/impulsivity subscale of the ADHD-SR in adulthood (Kooij et al., 2005).

^g^ Participants gave a rating (1-5) on their perceived health on the Health Questionnaire (Central Bureau of Statistics (CBS), 1989).

^h^ Participants gave an age-relative rating (1-5) on their perceived health compared to others of their age on the HQ (Central Bureau of Statistics (CBS), 1989).

^i^ Participants rating on perceived health on the World Health Organization Quality of Life questionnaire (WHO-QoL; Whoqol Group, 1998)

^j^ Participants subscore on the subscale physical health of the WHO-QoL questionnaire (Whoqol Group, 1998).

| **Condition** | **Autism** | **No autism** | **OR** | **CI** | **χ^2^** | **Adj OR** | **Adj CI** | **Adj χ^2^** |
| --- | --- | --- | --- | --- | --- | --- | --- | --- |
| Anxiety disorders | 32 (16.8%) | 4 (2.1%) | 8.54 | 3.43-27.01 | 26.32** | 7.7 | 3.08-24.41 | 23.09** |
| Mood disorders | 70 (36.6%) | 6 (3.1%) | 16.65 | 7.75-42.31 | Inf** | 18.78 | 8.52-48.74 | Inf** |
| Personality disorders | 30 (15.7%) | 1 (0.5%) | 24.11 | 6.26-216.47 | 34.8** | 25.4 | 6.51-229.29 | 34.93** |
| Alcohol addiction | 6 (3.1%) | 0 (0%) | 13.49 | 1.58-1763.98 | 6.23* | 10.43 | 1.22-1361.09 | 4.81* |
| Drug addiction | 6 (3.1%) | 0 (0%) | 13.49 | 1.58-1763.98 | 6.23* | 11.09 | 1.15-1515.85 | 4.43* |
| Eating disorders^a^ | 2 (1%) | 0 (0%) | 5.08 | 0.41-702.35 | 1.48 | 7.4 | 0.59-1015.28 | 2.31 |
| Post-traumatic stress disorder^a^ | 8 (4.2%) | 0 (0%) | 17.83 | 2.2-2311.97 | 8.83** | 16 | 1.95-2076.92 | 7.87** |
| Obsessive compulsive disorder^a^ | 2 (1%) | 0 (0%) | 5.08 | 0.41-702.35 | 1.48 | 6.38 | 0.51-881.27 | 1.95 |
| Other^b^ | 8 (4.2%) | 1 (0.5%) | 5.91 | 1.31-55.93 | 5.58* | 5.46 | 1.21-51.69 | 4.99* |

**Table S2.** Occurrence rates and (Adjusted) Odds Ratios of MHCs in our 50+ Subsample.

**Table S3.** Occurrence rates and (Adjusted) Odds Ratios of PHCs in our 50+ Subsample.

| **Condition** | | **Autism** | **No autism** | **OR** | **CI** | **χ^2^** | **Adj OR** | **Adj CI** | **Adj χ^2^** |
| --- | --- | --- | --- | --- | --- | --- | --- | --- | --- |
| Respiratory | Total^a^ | 21 (11%) | 10 (5.2%) | 1.38 | 0.81-2.4 | 1.39† | 2.12 | 0.99-4.8 | 3.76 |
|  | Asthma | 12 (6.3%) | 8 (4.2%) | 1.15 | 0.63-2.14 | 0.21 | 1.39 | 0.57-3.58 | 0.52 |
|  | COPD | 11 (5.8%) | 3 (1.6%) | 2.27 | 0.88-6.72 | 2.86† | 3.48 | 1.11-14.07 | 4.63† |
| Cardiovascular | Total | 55 (28.8%) | 71 (37%) | 0.74 | 0.51-1.07 | 2.59 | 0.79 | 0.5-1.24 | 1.06 |
|  | Heart attack | 22 (11.5%) | 30 (15.6%) | 0.66 | 0.39-1.12 | 2.36 | 0.79 | 0.42-1.46 | 0.57 |
|  | CVA/TIA | 8 (4.2%) | 3 (1.6%) | 2.82 | 0.92-11.16 | 3.28 | 3.37 | 0.97-14.69 | 3.62 |
|  | Blood pr. | 37 (19.4%) | 46 (24%) | 0.71 | 0.46-1.11 | 2.2 | 0.86 | 0.52-1.41 | 0.37 |
|  | Vascular dis. | 7 (3.7%) | 7 (3.6%) | 1.41 | 0.57-3.72 | 0.56 | 1.21 | 0.41-3.57 | 0.12 |
|  | Diabetes | 18 (9.4%) | 13 (6.8%) | 1.58 | 0.83-3.13 | 1.89 | 1.54 | 0.73-3.32 | 1.29 |
| Rheumatic | Total | 69 (36.1%) | 69 (35.9%) | 1.2 | 0.84-1.71 | 1.04 | 1.18 | 0.76-1.82 | 0.54 |
|  | Fibromyalgia | 6 (3.1%) | 7 (3.6%) | 1.56 | 0.68-3.82 | 1.07 | 0.85 | 0.27-2.64 | 0.08 |
|  | SLE | 0 (0%) | 1 (0.5%) | 0.28 | 0-5.24 | 0.72 | 0.27 | 0-4.95 | 0.76 |
|  | Arthritis | 64 (33.5%) | 64 (33.3%) | 1.01 | 0.7-1.46 | <.01 | 1.19 | 0.77-1.86 | 0.61 |
| Cancer |  | 20 (10.5%) | 18 (9.4%) | 0.95 | 0.52-1.72 | 0.03 | 1.38 | 0.69-2.78 | 0.85 |
| Ulcer |  | 7 (3.7%) | 7 (3.6%) | 1.65 | 0.69-4.27 | 1.25 | 1.27 | 0.42-3.9 | 0.19 |
| Bowel | Total | 48 (25.1%) | 23 (12%) | 2.53 | 1.66-3.92 | 19.45** | 2.63 | 1.52-4.66 | 12.16** |
|  | IBS | 21 (11%) | 9 (4.7%) | 2.25 | 1.28-4.11 | 8.13† | 2.39 | 1.09-5.59 | 4.8† |
|  | Obstipation | 28 (14.7%) | 6 (3.1%) | 4.07 | 2.17-8.28 | 21.26** | 5.86 | 2.49-15.96 | 18.19** |
|  | Crohn’s dis. | 1 (0.5%) | 1 (0.5%) | 0.5 | 0.05-3.79 | 0.46 | 1.14 | 0.09-14.67 | 0.01 |
| Liver | Total | 3 (1.6%) | 2 (1%) | 1.86 | 0.44-10.41 | 0.7 | 1.19 | 0.23-7.22 | 0.04 |
|  | Cirrhosis | 0 (0%) | 1 (0.5%) | 0.28 | 0-5.24 | 0.72 | 0.37 | 0-6.72 | 0.44 |
|  | Hepatitis | 1 (0.5%) | 1 (0.5%) | 1.4 | 0.19-15.37 | 0.11 | 0.82 | 0.07-10.17 | 0.03 |
| Epilepsy |  | 3 (1.6%) | 1 (0.5%) | 3.69 | 0.77-35.52 | 2.58 | 2.5 | 0.4-26 | 0.95 |
| Allergy | Total | 88 (46.1%) | 46 (24%) | 2.52 | 1.79-3.56 | 28.91** | 2.58 | 1.67-4.02 | 18.54** |
|  | Hay fever | 38 (19.9%) | 31 (16.1%) | 1.46 | 0.99-2.17 | 3.6 | 1.23 | 0.73-2.09 | 0.58 |
|  | Eczema | 42 (22%) | 17 (8.9%) | 2.36 | 1.5-3.8 | 14.24** | 2.8 | 1.55-5.23 | 12.02** |
| Thyroid | Total | 16 (8.4%) | 8 (4.2%) | 2.26 | 1.08-5.1 | 4.73 | 2.82 | 1.16-7.43 | 5.24† |
|  | Graves dis. | 0 (0%) | 1 (0.5%) | 0.28 | 0-5.24 | 0.72 | 0.9 | 0.01-95.42 | <.01 |
|  | Hypothyroid | 12 (6.3%) | 4 (2.1%) | 3.1 | 1.26-8.96 | 6.28† | 3.99 | 1.34-14.26 | 6.29† |
|  | Hyperthyroid | 1 (0.5%) | 1 (0.5%) | 0.84 | 0.07-10.36 | 0.02 | 0.89 | 0.07-10.98 | 0.01 |
| Auto immune conditions^b^ | |  | 1 (0.5%) | 4.27 | 0.93-40.63 | 3.42 | 3.02 | 0.59-29.68 | 1.67 |
| Headache / Migraine^b^ | |  | 2 (1%) | 1.46 | 0.52-4.55 | 0.5 | 1.4 | 0.26-8.63 | 0.16 |
| Neurological & CNS disease^b^ | |  | 4 (2.1%) | 1.03 | 0.29-3.85 | <.01 | 0.55 | 0.09-2.59 | 0.56 |
| Sleep disorders^b^ | |  | 0 (0%) | 3.69 | 0.77-35.52 | 2.58 | 9.54 | 0.99-1274.73 | 3.78 |
| Nutrition deficiencies^b^ | |  | 0 (0%) | 2.52 | 0.13-368.65 | 0.36 | 3.12 | 0.16-494.33 | 0.52 |
| Other^c^ | |  | 15 (7.8%) | 2.52 | 1.47-4.52 | 11.59† | 2.31 | 1.21-4.57 | 6.46† |

*Note*. OR, odds ratio; CI, confidence interval; Adj, adjusted; pr, pressure; dis, disease; Inf, infinite; *=Corrected *p*<.05; **= Corrected *p*<.01. †=Differences did not persist after correcting for family wise errors (Benjamini & Hochberg, 1995).

^a^ Total classifications included the specific conditions that are specified as sub-conditions, but also included other unspecified/unknown conditions within that category.

^b^ These conditions/diseases were not specifically asked but often named spontaneously.

^c^ Other conditions that were spontaneously named varied widely and could not be categorized.


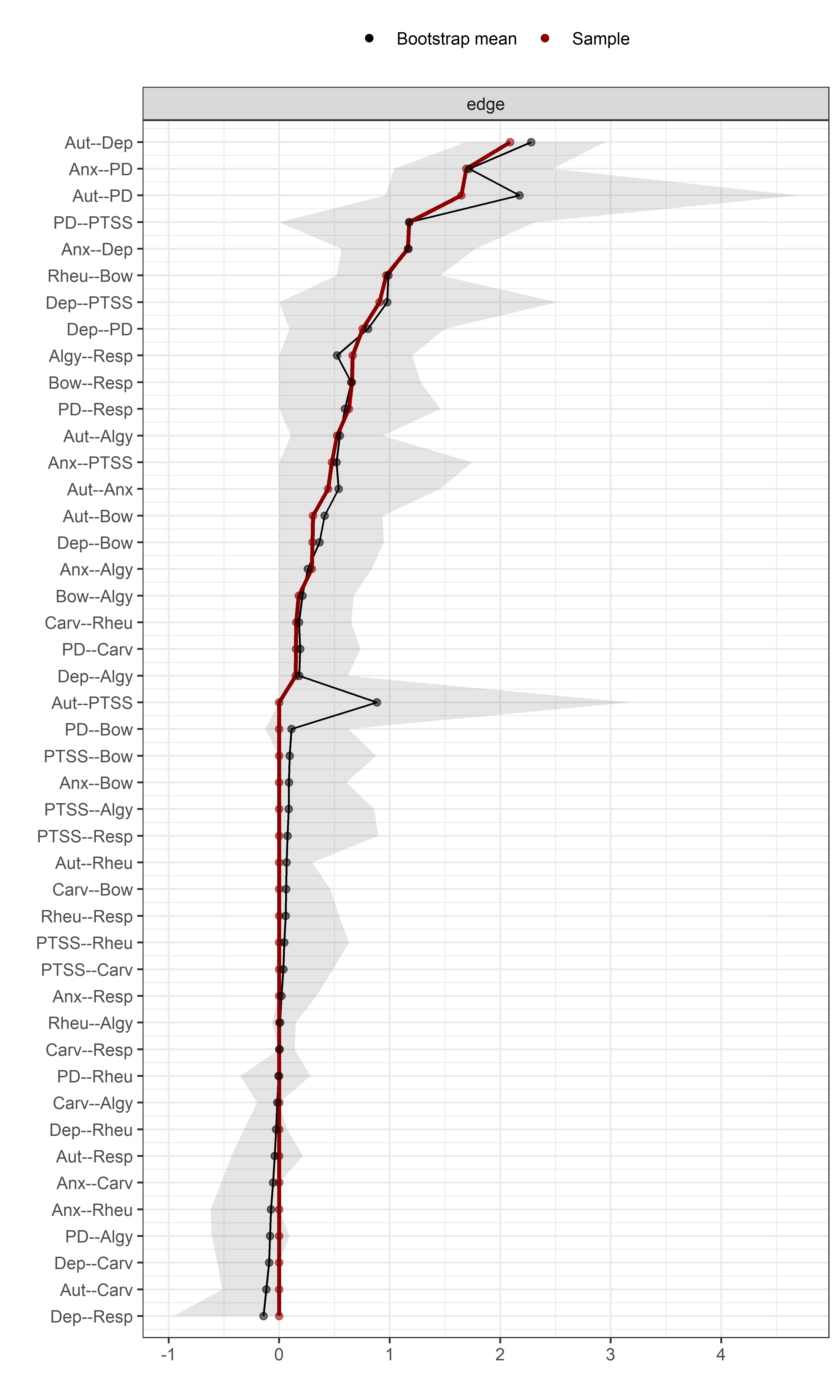


**Figure S1.** Bootstrapped edges weights.

*Note.* All potential edges in the network, ordered from the highest edge to the lowest edge weights in the sample, are displayed on the y-axis. Edge weights are displayed on the x-axis. The red dots indicate the edge weights in the sample, whereas the black dots indicate the mean in the bootstrapped samples. Bootstrapped confidence intervals are in grey. Abbreviations of all edges are provided in the legend of Figure S4.


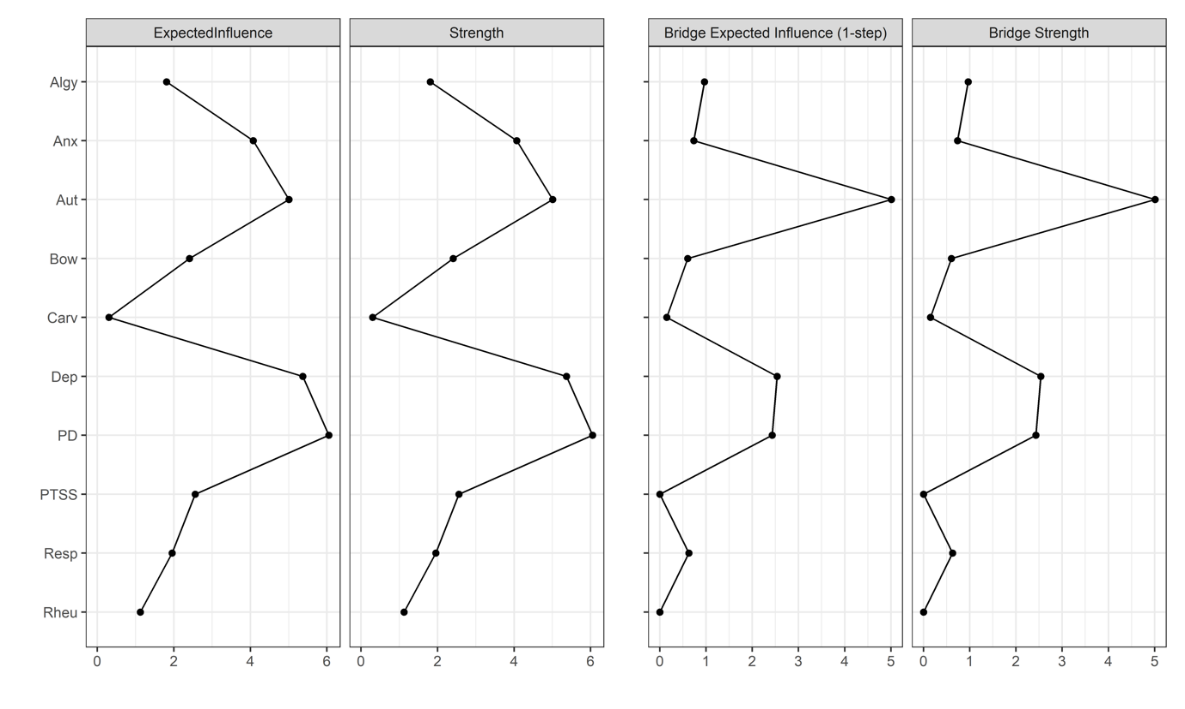


**Figure S2.** Centrality indices.

*Note*. Nodes are on the y-axis and displayed in alphabetical order. On the x-axis the (bridge) expected influence and (bridge) strength of each node is provided. In our network, expected influence and strength are equivalent as no significant negative edges were present in our network.

Bridge expected influence and strength represented the strength of the connections between three pre-specified clusters: autism, MHC and PHC. Note that in the case of autism, all edges were taken into account because this cluster only contained the autism node. Therfore, bridge sterngth/expected influence is not very informative for this particular node. For all other nodes, it gives an indication of across-cluster edges (e.g., an MHC node (PD) with all PHCs and autism).

Abbreviations of all nodes are provided in the legend of Figure S4.


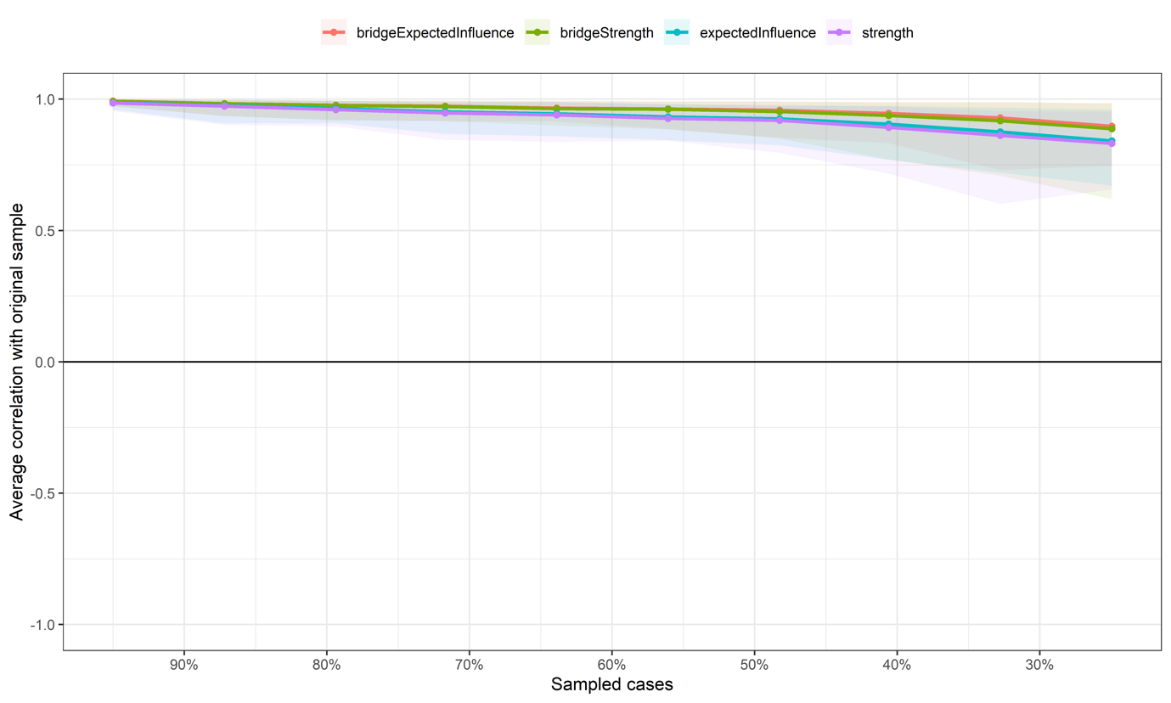


**Figure S3.** Case-drop bootstrapping of the centrality indices.

*Note.* Case-drop bootstrapping provides an estimate of the stability of our centrality indices by taking a bootstrapped sample of a certain percentage of the original sample. The percentage of dropped cases is provided on the x-axis, meaning that for instance 50% of the original sample was used for a bootstrap at 50%. The average correlation of the centrality indices between the dropped cases sample and original sample is provided on the y-axis. Our analysis indicates that the correlation between the original sample and the dropped cases sample remains above .80, even when only 30% of the original cases are sampled. This indicates excellent stability of all of the centrality indices, as a minimum of .25 at 70% needs to reached to be sufficient, or .50 to indicate good stability (Epskamp, 2018b).


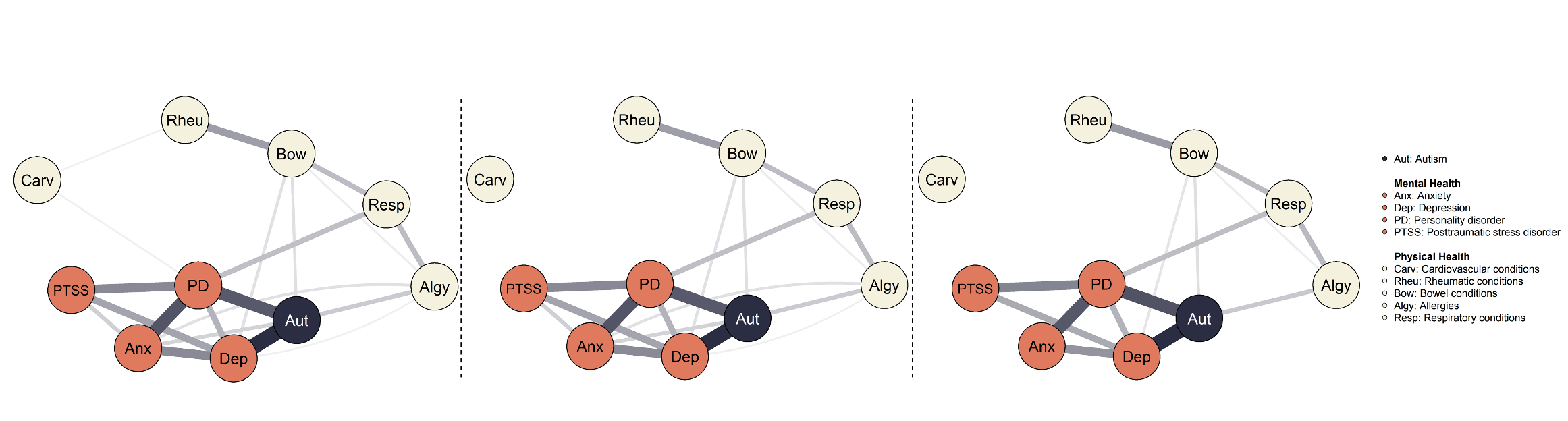


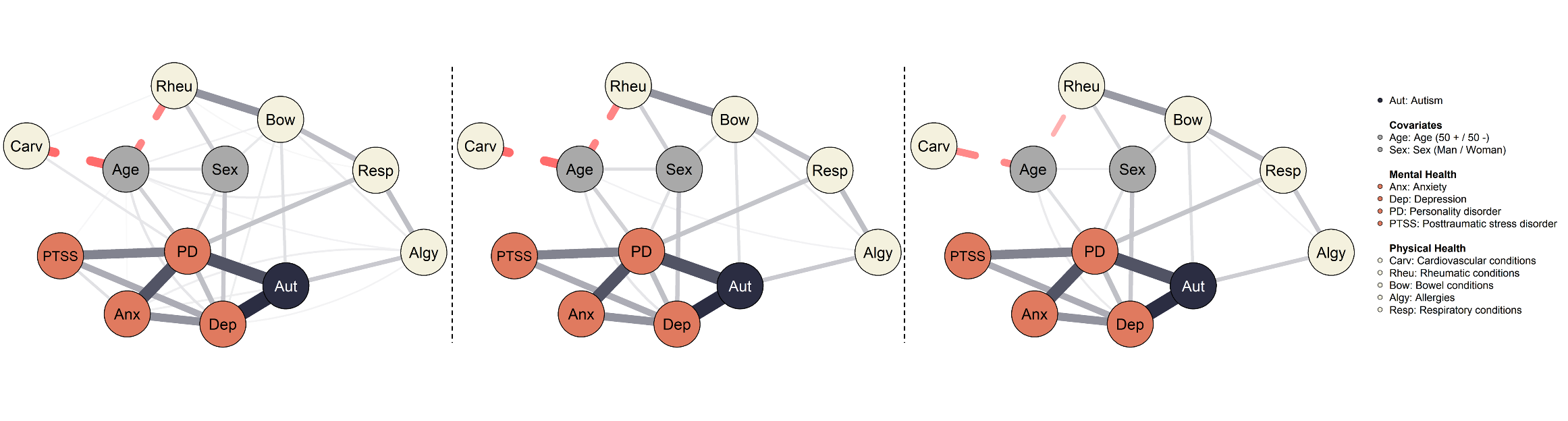
**Figure S4.** Sensitivity analyses of the network.

**Figure S5.** Sensitivity analyses of the covariate network.

*Note.* The panels represent the network (S4) and covariate network (S5) with different parameter settings, with the left being least conservative (*γ*=0 + OR rule), middle being somewhat conservative (*γ*=0 + AND rule), and the right being most conservative (*γ*=.25 + AND rule). It can be seen that although some of the weaker edges disappear, most edges remain present throughout all settings. The left panels are equivalent to Figure 2, and are only displayed here to ease comparison with the other networks.


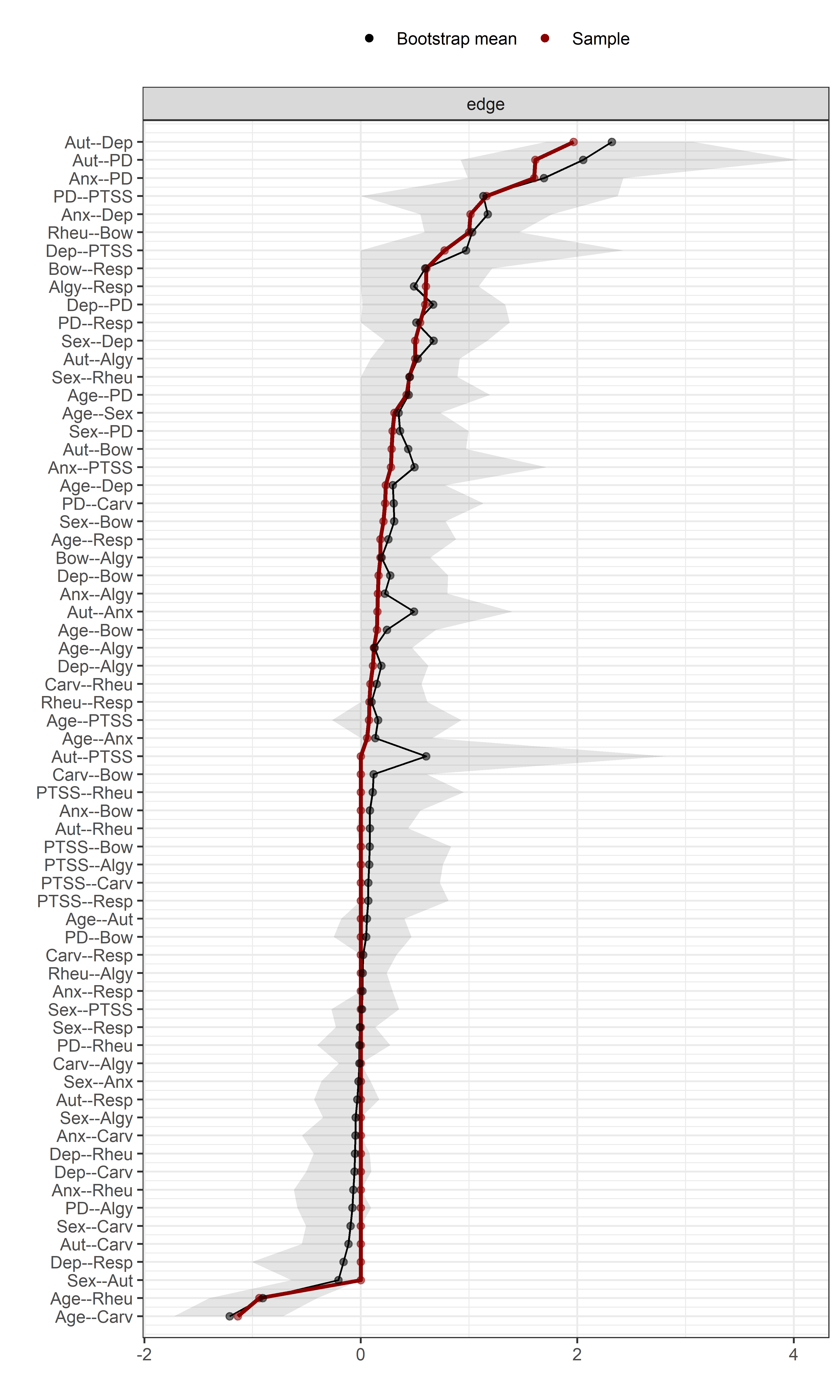


**Figure S6.** Bootstrapped edges weights of the covariate network.

*Note.* Please see Figure S1 for additional information. Abbreviations of all edges are provided in the legend of Figure S5.


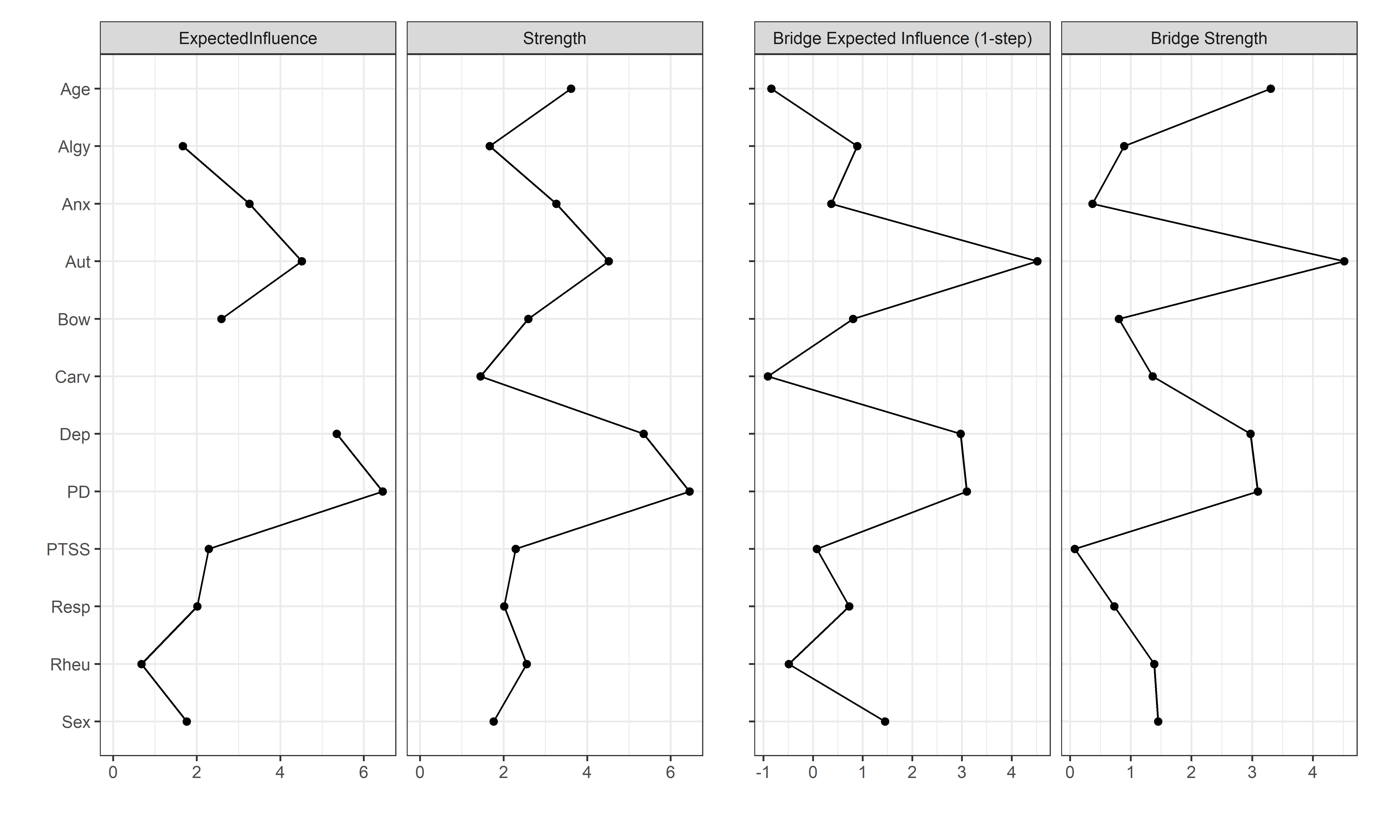


**Figure S7.** Centrality indices of the covariate network.

*Note.* As expected influence takes the directionality of the effects into account, expected influence is negative (i.e., not displayed) for some nodes (Age, Carv).

Please see Figure S2 for additional information. Abbreviations of all nodes are provided in the legend of Figure S5.


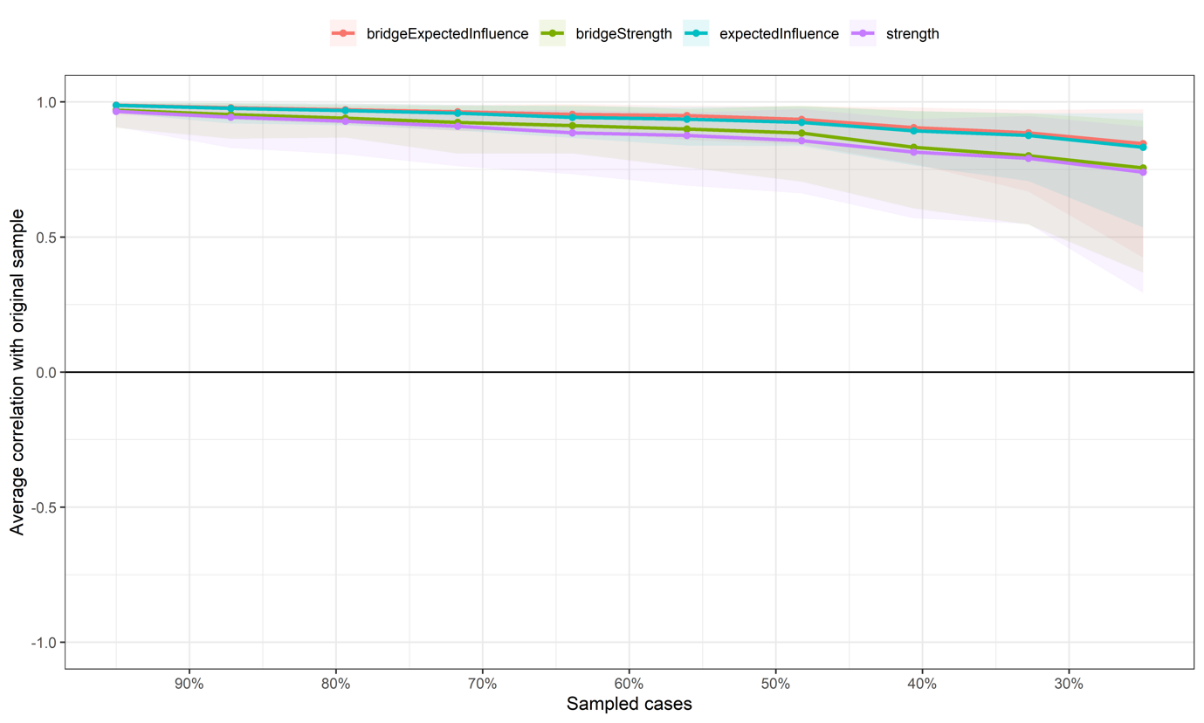


**Figure S8.** Case-drop bootstrapping of the centrality indices of the covariate network.

*Note*. All of the centrality indices of the covariate network are very stable according to this bootstrap analysis (.75 at 30%). Please see Figure S3 for additional information.
